# Evaluation of the Prognostic Value of STEAP1 in Lung Adenocarcinoma and Insights Into Its Potential Molecular Pathways via Bioinformatic Analysis

**DOI:** 10.3389/fgene.2020.00242

**Published:** 2020-03-20

**Authors:** Qiang Guo, Xi-xian Ke, Zhou Liu, Wei-Long Gao, Shi-Xu Fang, Cheng Chen, Yong-Xiang Song, Hao Han, Hong-Ling Lu, Gang Xu

**Affiliations:** ^1^Department of Thoracic Surgery, Affiliated Hospital of Zunyi Medical University, Zunyi, China; ^2^Department of Cardiac Surgery, Affiliated Hospital of Guizhou Medical University, Guiyang, China; ^3^Department of Biochemistry, Zunyi Medical University, Zunyi, China

**Keywords:** STEAP1, LUAD, Kyoto Encyclopedia of Genes and Genomes, The Cancer Genome Atlas, gene set enrichment analysis

## Abstract

**Background:**

Upregulation of the six-transmembrane epithelial antigen of prostate-1 (STEAP1) is closely associated with prognosis of numerous malignant cancers. However, its role in lung adenocarcinoma (LUAD), the most common type of lung cancer, remains unknown. This study aimed to investigate the role of STEAP1 in the occurrence and progression of LUAD and the potential mechanisms underlying its regulatory effects.

**Methods:**

STEAP1 mRNA and protein expression were analyzed in 40 LUAD patients via real-time PCR and western blotting, respectively. We accessed the clinical data of 522 LUAD patients from The Cancer Genome Atlas (TCGA) and the Gene Expression Omnibus (GEO) to investigate the expression and prognostic role of STEAP1 in LUAD. Further, we performed gene ontology (GO) analysis, Kyoto Encyclopedia of Genes and Genomes (KEGG) analysis, and gene set enrichment analysis (GSEA) to elucidate the potential mechanism underlying the role of STEAP1 in LUAD. The protein-protein interaction (PPI) network of STEAP1 was analyzed using the Search Tool for the Retrieval of Interacting Genes (STRING) database, and hub genes with significant positive and negative associations with STEAP1 were identified and their role in LUAD prognosis was predicted.

**Results:**

STEAP1 was significantly upregulated in LUAD patients and associated with LUAD prognosis. Further, TCGA data indicated that STEAP1 upregulation is correlated with the clinical prognosis of LUAD. GO and KEGG analysis revealed that the genes co-expressed with STEAP1 were primarily involved in cell division, DNA replication, cell cycle, apoptosis, cytokine signaling, NF-kB signaling, and TNF signaling. GSEA revealed that homologous recombination, p53 signaling pathway, cell cycle, DNA replication, apoptosis, and toll-like receptor signaling were highly enriched upon STEAP1 upregulation. Gene Expression Profiling Interactive Analysis (GEPIA) analysis revealed that the top 10 hub genes associated with STEAP1 expression were also associated with the LUAD prognosis.

**Conclusion:**

STEAP1 upregulation potentially influences the occurrence and progression of LUAD and its co-expressed genes via regulation of homologous recombination, p53 signaling, cell cycle, DNA replication, and apoptosis. STEAP1 is a potential prognostic biomarker for LUAD.

## Introduction

Lung cancer has one of the highest rates of incidence and mortality among all types of malignant tumors (Bray et al., 2018). Non-small cell lung cancer (NSCLC) accounts for 85% of all lung cancers (El-Zein et al., 2017; Colling et al., 2019). Lung adenocarcinoma (LUAD), the most common type of NSCLC and accounting for approximately 40% of all lung cancers, is one of the leading causes of respiratory cancer-associated deaths (Maemura et al., 2018; Kleczko et al., 2019). With the use of highly sensitive low dose computerized tomography (CT) to detect lung cancer, and an increase in the overall awareness about the prevention and diagnosis of early NSCLC, the prognosis of NSCLC patients has significantly improved (Hasan et al., 2014; Kassem et al., 2019). Nevertheless, owing to the insidious nature of NSCLC progression, most of the patients still present at an advanced stage upon diagnosis. Consequently, most of these patients cannot be surgically treated and respond poorly to radiotherapy and chemotherapy, resulting in a <15% chance of 5-year survival (Molina et al., 2008; Li et al., 2018). The advent of targeted therapy holds promise among NSCLC patients, especially among LUAD patients; however, the poor prognosis still remains a concern. Therefore, it is important to identify new biomarkers for early stratification and diagnosis of high-risk lung cancer patients with a poor prognosis.

Six-transmembrane epithelial antigen of prostate-1 (STEAP1), also known as PRSS24, belonging to the STEAP family, is a cell surface antigen overexpressed in various tumors and is closely associated with the prognosis of numerous malignant tumors (Grunewald et al., 2012a; Ihlaseh-Catalano et al., 2013; Lee et al., 2016; Barroca-Ferreira et al., 2018). STEAP1 is upregulated in prostate cancer and associated with a high Gleason score, seminal vesicle invasion, and biochemical recurrence (BCR) (Ihlaseh-Catalano et al., 2013). Targeting STEAP1 expression may potentially inhibit the proliferation of and induced apoptosis in prostate cancer cells (Gomes et al., 2014, 2018). However, STEAP1 downregulation has been reported in endometrial carcinoma, and STEAP1 upregulation is associated with a better patient prognosis rather than STEAP1 downregulation (Sun et al., 2019). In contrast, some studies have indicated that STEAP1 upregulation is associated with reduced growth and migration of endometrial cancer cells (Sun et al., 2019). Furthermore, STEAP1 is reportedly upregulated in Ewing’s tumor and colorectal cancer, and the prognosis of patients with high STEAP1 expression levels was better than that of patients with low expression levels (Grunewald et al., 2012c; Lee et al., 2016; Nakamura et al., 2019). STEAP1 is aberrantly expressed in multiple tumors and is associated with the occurrence, development, and prognosis of malignant tumors, indicating that STEAP1 might be a potential diagnostic and therapeutic marker for cancers. In addition, Zhuang et al. (2015) first reported that STEAP1 is upregulated in lung cancer tissues. However, the role and potential value of STEAP1 in LUAD has not been reported. This study aimed to analyze the expression and clinical significance of STEAP1 in LUAD and to explore the role of STEAP1 in the occurrence and progression of LUAD and the potential mechanism underlying its regulatory functions.

## Materials and Methods

### Clinical Samples

Tumor and tumor-adjacent normal tissue (5 cm proximity) specimens were obtained from 40 LUAD patients, who received surgical treatment at the Department of Thoracic Surgery, Affiliated Hospital of Zunyi Medical University from April 2018 to March 2019. The clinical characteristics of the 40 patients are shown in Table 1. This study was approved by the Ethics Committee of Affiliated Hospital of Zunyi Medical University. All of the patients provided written informed consent, and no other special treatment was administered before surgery.

**TABLE 1:** Clinicopathological characteristics of the 40 LUAD patients.

Clinical characteristics	Total (*N* = 40)	Percentage (%)
Age at diagnosis (years)	58.5 (42–78)	
**Gender**		
Female	25	62.5
Male	15	37.5
**Clinical stage**		
I	21	52.5
II	14	35
III	4	10
IV	1	2.5
**T stage**		
1	11	27.5
2	25	62.5
3	3	7.5
4	1	2.5
**Lymph nodes**		
0	19	47.5
1	12	30
2	7	17.5
3	2	5
**Distant metastasis**		
Negative	39	97.5
Positive	1	2.5

### Quantitative Real-Time PCR Analysis

Total RNA was extracted from tissues, using Trizol reagent (Invitrogen, United States) and transcribed to cDNA by using the reverse transcription kit (Takara, Japan) in accordance with the manufacturer’s instructions. Real-time PCR was then carried out with *GAPDH* as the internal control, using the following primers: STEAP1: 5′-GGCGATCCTACAG ATACAAGTTGC-3′ (forward); 5′-CCAATC CCACAATTCCCAGAGAC-3′ (reverse); GAPDH: 5′-AACGGATTTGGTCGTATTG-3′ (forward); 5′-GGAAGATGGTGATGGGATT-3′ (reverse) (Feng et al., 2013; Gomes et al., 2013). This experiment was performed in triplicate.

### Western Blotting

Total protein was extracted from tissues and quantified using the BCA kit (Solarbio, China) in accordance with the manufacturer’s instructions. Extracted proteins were then resolved via SDS-PAGE (10% resolving gel) and electro-transferred onto a polyvinylidene difluoride membrane. The membranes were blocked with 5% skimmed milk on the shaker for 1 h and subsequently probed with a rabbit anti-human STEAP1 antibody (1:1000, HuaBio, China). Protein bands were probed using HRP-labeled secondary antibody (1:5000, Bioss, China). Tubulin was used as the internal control. Immunoblots were visualized using a Gel doc imaging system (Bio-Rad, China). This experiment was performed in triplicate.

### Data Curation and Bioinformatics Analysis

In May 2019, the gene expression data of 535 LUAD tissues and 59 normal lung tissues with the type of HTSeq-FPKM, and the clinical data of 522 LUAD patients were downloaded from TCGA^1^ (Table 2). Among the 594 samples in TCGA, there were 57 paired LUAD tissues and adjacent normal lung tissues. Furthermore, STEAP1 expression data were downloaded from the data sets of GSE2514 and GSE10072 in the Gene Expression Omnibus (GEO) database. The clinicopathological parameters and prognostic data of the patients in TCGA were used to screen and the cases with unknown or incomplete parameters and lack of prognostic follow-up data were excluded.

**TABLE 2:** Clinicopathological characteristics of the 522 LUAD patients from TCGA database.

Clinical characteristics	Total (*N* = 522)	Percentage (%)
Age at diagnosis (years)	65.3 (33–88)	
**Gender**		
Female	280	53.6
Male	242	46.4
**Clinical stage**		
I	279	54.3
II	124	24.1
III	85	16.5
IV	26	5.1
**T stage**		
1	172	33.1
2	281	54.1
3	47	9.1
4	19	3.7
**Lymph nodes**		
0	335	65.7
1	98	19.2
2	75	14.7
3	2	0.4
**Distant metastasis**		
Negative	353	93.4
Positive	25	6.6

### Screening of STEAP1 Co-expressed Genes

R (v.3.5.2) was used to screen the LUAD transcriptome expression matrix from TCGA databases to identify the genes co-expressed with STEAP1. Pearson’s correlation coefficient (*r*) was used to investigate the correlation between STEAP1 and co-expressed genes (Wang et al., 2015). |*r*| > 0.35 and *P* < 0.001 indicated a moderate correlation between STEAP1 with co-expressed gene.

### Gene Ontology and Kyoto Encyclopedia of Genes and Genomes Analysis

To investigate the biological role of STEAP1 and its mechanisms regulating LUAD progression, we performed gene ontology (GO) annotation and Kyoto Encyclopedia of Genes and Genomes (KEGG) analysis for genes co-expressed with STEAP1 as previously reported (Zhou et al., 2019). GO analysis was performed with the terms of biological process (BP), molecular function (MF), and cellular component (CC). STEAP1 co-expressed genes were imported into the Metascape database and the species was set to *Homo sapiens* (*P* < 0.05) to determine the BP, MF, and CC, and KEGG pathway analysis was conducted as previously described (Wang L. et al., 2019). Gene set enrichment analysis (GSEA) is performed to explore gene expression data from basic data. Gene expression data in TCGA was divided into high and low expression groups in accordance with the median expression level of STEAP1 to investigate the effect of different levels of STEAP1 expression on each gene to explore the mechanism underlying the role of STEAP1 in LUAD progression. Each analysis arranged the genome 1,000 times and STEAP1 expression levels were considered phenotypic markers. Furthermore, nominal *P*-values and enrichment scores (NES) were considered to classify enrichment pathways for each phenotype as previously described (Wu and Zhang, 2018).

### Construction of Protein-Protein Interaction Network and Identification of Hub Genes

Search Tool for the Retrieval of Interacting Genes (STRING), a functional protein association networks database^2^ is an online database to analyze the interactions among multiple genes and generate network relationships. Herein, to explore the role of STEAP1 co-expressed genes, the online database of STRING was applied to analyze associations among the Protein-Protein Interaction (PPI) network of STEAP1 co-expressed genes in LUAD, and the species was set to *Homo sapiens* and a combined score >0.7 was considered statistically significant (Szklarczyk et al., 2015; Zhang et al., 2020). Furthermore, the PPI network thus generated was imported into Cytoscape 3.6.1 software and the top 10 genes were screened out as the hub genes, using the CytoHubba plug-in to further assess the biological function of STEAP1 in accordance with the value of hub genes (Yang et al., 2019). The online tool Gene Expression Profiling Interactive Analysis (GEPIA)^3^ was used to analyze the expression of hub genes and their effect on the prognosis of LUAD patients (Tang et al., 2017).

### Statistical Analysis

Perl and R were used for data processing and statistical analysis. STEAP1 expression and its association with the clinicopathological features of patients were analyzed via Wilcoxon signed-rank test and logistic regression analysis. Cox regression analysis and the Kaplan–Meier test were performed to analyze the correlation between clinicopathological features and the overall survival (OS). Genes co-expressed with STEAP1 were screened via Pearson’s correlation analysis.

## Results

### STEAP1 Was Highly Expressed in LUAD Tissues

**FIGURE 1:**
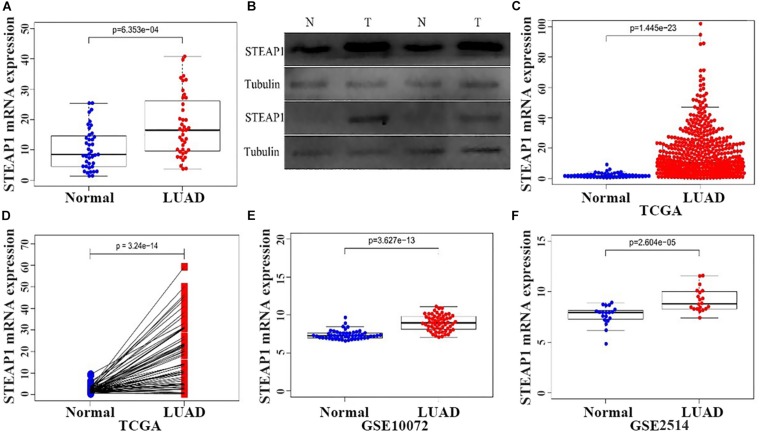
Increased expression of STEAP1 in LUAD. **(A)** qRT-PCR was performed to detect the expression of STEAP1 mRNA in tumor and adjacent tissues of 40 LUAD patients. **(B)** The protein expression of STEAP1 in tumor and adjacent tissues of 40 LUAD patients was detected visa western blotting. **(C)** STEAP1 mRNA levels in LUAD tissues in TCGA database. **(D)** STEAP1 mRNA expression levels in tumor and adjacent tissues of 57 patients with LUAD in TCGA database. **(E,F)** STEAP1 mRNA levels in LUAD tissues in GEO database (GSE2514 and GSE10072). The data are indicated as mean ± standard deviation (SD). Normal, Normal lung tissue; LUAD, lung adenocarcinoma tissue; TCGA, The Cancer Genome Atlas; GEO, Gene Expression Omnibus.

STEAP1 expression was analyzed via qRT-PCR and western blotting in 40 tumor and tumor-adjacent normal tissue specimens from LUAD patients (Figures 1A,B). The present results show that among the 40 samples, STEAP1 was significantly upregulated in 31 cases (75%) and 34 cases (85%) of LUAD patients, respectively (*P* < 0.05). Assessment of 535 specimens of LUAD tissue and 59 tumor-adjacent normal tissue specimens in TCGA database revealed that STEAP1 was significantly upregulated in LUAD tissues rather than in the adjacent non-cancerous tissues (Figure 1C, *P* < 0.05). Moreover, we analyzed STEAP1 expression in 57 pairs of matched specimens of tumor tissue and tumor-adjacent normal tissue from TCGA database from one patient (Figure 1D, *P* < 0.05). GEO analysis (GSE2514 and GSE10072) confirmed that STEAP1 was significantly upregulated in LUAD tissues (Figures 1E,F, *P* < 0.05).

### STEAP1 Upregulation Was Correlated With the Clinical Stage, T Stage, and LNM in LUAD Patients

**FIGURE 2:**
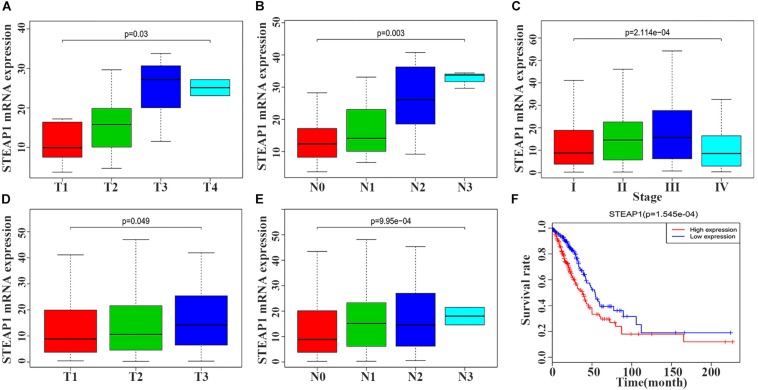
Six-transmembrane epithelial antigen of prostate-1 (STEAP1) expression levels were associated with the clinicopathological characteristics and prognosis of LUAD patients. **(A,B)** The expression level of STEAP1 was associated with T stage and lymph node metastasis in 40 LUAD patients. **(C–E)** The expression level of STEAP1 was associated with clinical stage, T staging and lymph node metastasis for LUAD patients in TCGA database. **(F)** LUAD patients with high STEAP1 expression in TCGA database had a lower overall survival than patients with low STEAP1 expression. T, T Stage; N, Lymph node metastasis.

STEAP1 upregulation in the tumor tissue specimens of 40 LUAD patients significantly correlated with an advanced T stage and lymph node metastasis (LNM) (Figures 2A,B, *P* < 0.05). Furthermore, analysis of LUAD data from TCGA database revealed a significant correlation between STEAP1 upregulation and an advanced clinical stage, T stage, and LNM (Figures 2C–E, *P* < 0.05). Univariate logistic regression analysis revealed that in LUAD patients from TCGA data, STEAP1 expression levels were correlated with the clinicopathological features of the patients (Table 3). STEAP1 upregulation was significantly correlated with the clinical stage (OR = 2.2; I vs. II, and OR = 2.4; I vs. III), TNM stage (OR = 1.5; T1 vs. T2, and OR = 2.0; T1 vs. T3), and LNM (OR = 2.2; N0 vs. N1, and OR = 2.3; N0 vs. N2). Together, these results suggest that LUAD patients with high STEAP1 expression levels were more likely to have an advanced LUAD stage and a poor prognosis than those with low STEAP1 expression levels.

**TABLE 3:** Relationship between STEAP1 expression and clinicopathological features from TCGA database analyzed by logistic regression analysis.

Clinical characteristics	Total (*N*)	Odds ratio in STEAP1 expression	*P*-Value
Age	494	0.99(0.97−1.008)	0.107
Gender	513	0.87(0.62−1.24)	0.450
**Clinical stage**			
I vs. II	395	2.18(1.41−3.39)	0.0005
I vs. III	358	2.36(1.44−3.95)	0.0008
**T stage**			
T1 vs.T2	444	1.53(1.04−2.26)	0.030
T1 vs. T3	215	2.01(1.05−3.94)	0.037
**Lymph nodes**			
N0 vs. N1	425	2.17(1.36−3.49)	0.001
N0 vs. N2	404	2.30(1.38−3.92)	0.002
Distant metastasis (positive vs. negative)	369	0.65(0.28−1.47)	0.310

### Survival Analysis and Multivariate Analysis

Kaplan–Meier survival analysis revealed that the prognosis of LUAD patients with high STEAP1 expression levels was worse than that of patients with low STEAP1 expression levels (Figure 2F, *P* < 0.001). Univariate analysis revealed that the clinical stage, T stage, and LNM significantly correlated with the overall survival of patients with LUAD (Table 4A), whereas multivariate analysis revealed that the clinical stage and LNM were independent factors for a poor prognosis among patients with LUAD (Table 4B).

**TABLE 4:** Associations between overall survival (OS) and clinicopathologic features of LUAD patients from TCGA database analyzed by cox regression **(A)**, and multivariate survival model after variable selection **(B)**.

Clinical characteristics	HR (95%CI)	*P*-Value
**A**		
Age (continuous)	1.00 (0.98 – 1.02)	0.929
Gender	1.00 (0.70 – 1.43)	0.996
Clinical stage	1.65 (1.40 – 1.94)	2.42E-09
TNM stage	1.62 (1.31 – 2.01)	9.57E-06
Lymph nodes	1.79 (1.46 – 2.19)	1.47E-08
Distant metastasis	1.53 (0.82 – 2.85)	0.181
STEAP1 expression (low vs. high)	1.01 (1.0 0– 1.01)	0.195
**B**		
Clinical stage	1.37 (1.09 – 1.73)	0.0007
T Stage	1.24 (0.98 – 1.55)	0.069
Lymph nodes	1.30 (1.00 – 1.69)	0.048

### Identification of STEAP1 Co-expressed Genes

**FIGURE 3:**
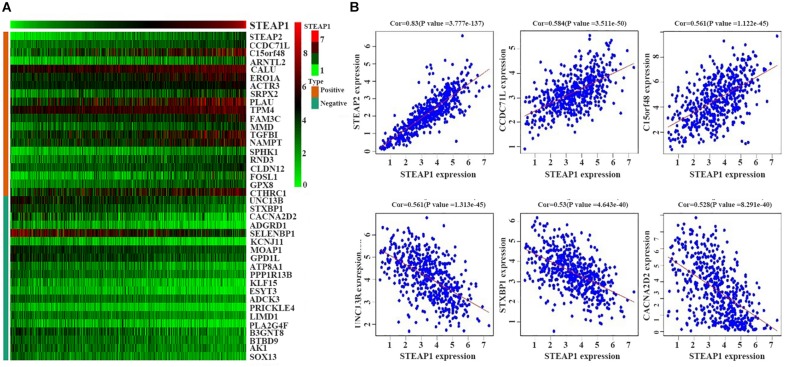
Positively and negatively correlated genes within STEAP1 co-expressed genes. **(A)** The heat map shows the top 20 positively and negatively related genes. **(B)** The top 3 positively and negatively related genes. Positive, positive correlation; Negative, negative correlation; Cor, correlation coefficient.

We selected 488 eligible genes co-expressed with STEAP1 in the LUAD transcriptional data downloaded from TCGA public database. Among these, 298 genes were positively associated with STEAP1 expression, while 190 genes were negatively associated with STEAP1 expression (Supplementary Table S1). A heat map was generated considering the top 20 genes positively and negatively correlated with STEAP1 expression (Figure 3).

### GO and KEGG Enrichment Analysis Revealed Pathways Regulated by STEAP1 in LUAD

**FIGURE 4:**
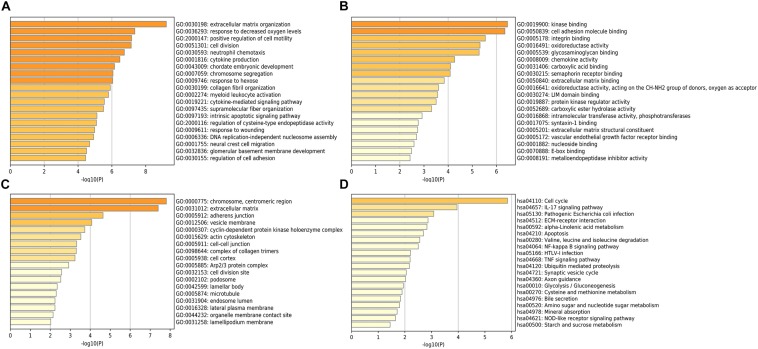
Analysis of STEAP1 co-expressed genes by GO and KEGG. **(A)** BP. **(B)** MF. **(C)** CC. **(D)** KEGG. GO, gene ontology; BP, biological process; MF, molecular functions; CC, cellular components; KEGG, Kyoto Encyclopedia of Genes and Genomes.

**FIGURE 5:**
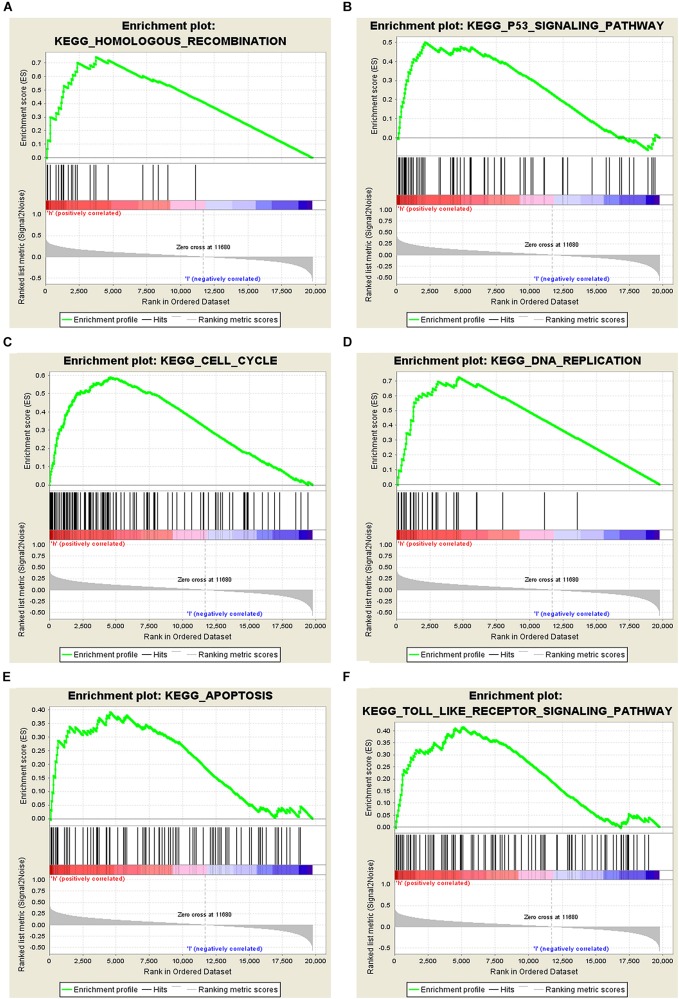
Analysis of related signaling pathways associated with the genes co-expressed with STEAP1 by GSEA. **(A)** Homologous recombination. **(B)** p53 signaling pathway. **(C)** Cell cycle. **(D)** DNA replication. **(E)** Apoptosis. **(F)** TOLL-like receptor signaling pathway. ES, enrichment score; NES, normalized ES; NOM *P*-value, normalized *P*-value.

The potential role of STEAP1 in LUAD progression was analyzed via GO and KEGG analysis. GO analysis included the analysis of biological process (BP), molecular function (MF), and cellular component (CC). We found that genes co-expressed with STEAP1 were primarily involved in cell adhesion, cell division, cytokine production, cytokine signaling, and DNA replication (Figures 4A–C). KEGG pathway analysis revealed that STEAP1 co-expressed genes were enriched in cell cycle regulation, the IL-17 signaling pathway, ECM receptor interaction, apoptosis, the NF-kB signaling pathway, and the TNF signaling pathway (Figure 4D). Furthermore, GSEA revealed that homologous recombination, the p53 signaling pathway, cell cycle, DNA replication, apoptosis, and the toll-like receptor signaling pathway were significantly enriched upon STEAP1 upregulation (Figure 5). Together, these results suggest that STEAP1 upregulation potentially regulates LUAD progression via homologous recombination, p53 signaling, cell cycle, DNA replication, and apoptosis.

### Potential Role of STEAP1-Related Hub Genes in Protein-Protein Interaction Network Equations

**FIGURE 6:**
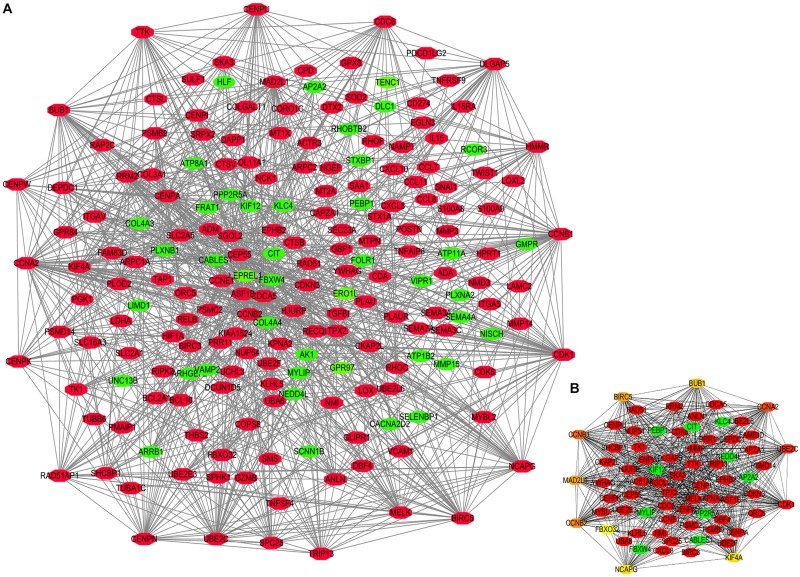
Protein-protein interaction (PPI) network constructed by STEAP1 co-expression genes. **(A)** PPI network. **(B)** Ten hub genes in PPI network. Red, positive correlation genes; green, negative correlation genes; orange and yellow, hub genes.

**FIGURE 7:**
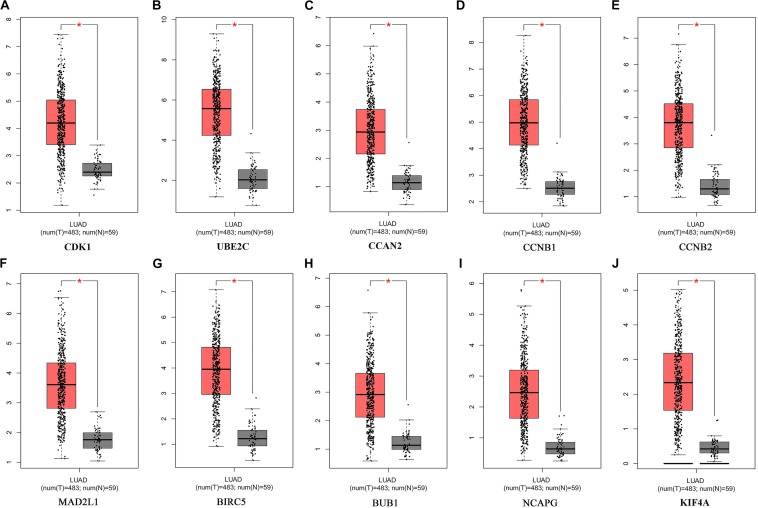
Expression of Hub genes of LUAD in the GEPIA database. **(A)** CDK1. **(B)** UBE2C. **(C)** CCAN2. **(D)** CCNB1. **(E)** CCNB2. **(F)** MAD2L1. **(G)** BIRC5. **(H)** BUB1. **(I)** NCAPG. **(J)** KIF4A. LUAD: lung adenocarcinoma; Red, lung adenocarcinoma tissue; Gray, normal lung tissue. *P < 0.05.

**FIGURE 8:**
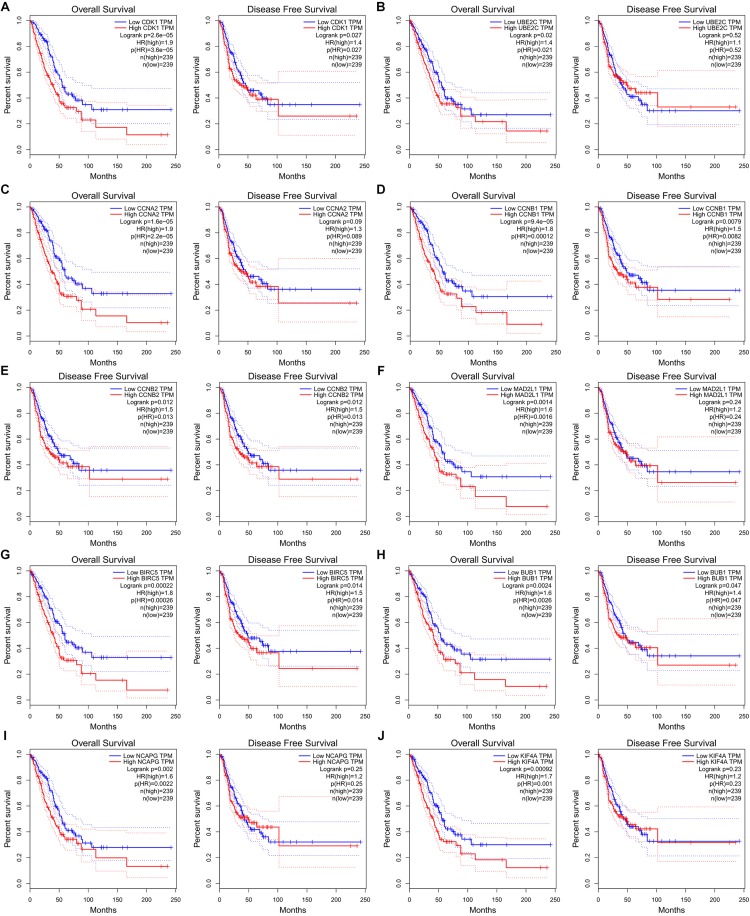
Ten hub genes are associated with overall survival (OS) and disease-free survival (DFS) in LUAD patients in GEPIA database. **(A)** CDK1. **(B)** UBE2C. **(C)** CCAN2. **(D)** CCNB1. **(E)** CCNB2. **(F)** MAD2L1. **(G)** BIRC5. **(H)** BUB1. **(I)** NCAPG. **(J)** KIF4A.

The potential biological function of STEAP1 was inferred by analyzing the roles of its co-expressed genes. A PPI network was constructed (Figure 6A) and the top 10 hub genes were identified on the basis of their degree of connectivity in the PPI network: *CDK1*, *UBE2C*, *CCNA1*, *CCNB1*, *CCNB2*, *MAD2L1*, *BIRC5*, *BUB1*, *NCAPG*, and *KIF4A* (Figure 6B and Table 5). The expression of the potential hub genes in LUAD and their prognostic values were analyzed via GEPIA. We found that these hub genes were upregulated in LUAD patients (Figure 7). While the 10 hub genes were associated with the OS of LUAD patients, the expression of *CDK1*, *CCNB1*, *CCNB2*, *BIRC5*, and *BUB1* was associated with disease-free survival (DFS) in LUAD patients (Figure 8).

**TABLE 5:** The top 10 hub genes in the protein-protein interaction (PPI) network.

Gene symbol	Gene description	Degree	*r*
CDK1	Cyclin-dependent kinases 1	51	0.377
UBE2C	Ubiquitin conjugating enzyme E2C	49	0.378
CCNA2	Cyclin A2	47	0.414
CCNB1	Cyclin B1	46	0.364
CCNB2	Cyclin B2	43	0.358
MAD2L1	MAD2 mitotic arrest deficient-like 1	41	0.423
BIRC5	Baculoviral inhibitor of apoptosis repeat-containing 5	41	0.389
BUB1	Budding uninhibited by benzimidazoles 1	40	0.356
NCAPG	Non-SMC condensin I complex subunit G	38	0.362
KIF4A	Kinesin family member 4A	38	0.359

## Discussion

Six-transmembrane epithelial antigen of prostate-1 is reportedly aberrantly regulated in numerous cancers, including prostate cancer, Ewing’s sarcoma, breast cancer and colorectal cancer, and is associated with the prognosis of cancer patients (Grunewald et al., 2012c; Ihlaseh-Catalano et al., 2013; Gomes et al., 2014; Lee et al., 2016; Barroca-Ferreira et al., 2018; Nakamura et al., 2019; Sun et al., 2019; Xie et al., 2019). Compared with tumor-adjacent normal tissues and benign prostatic hyperplasia (BPH) tissue, STEAP1 was upregulated in prostate cancer and prostatic intraepithelial neoplasia (PIN). Furthermore, STEAP1 expression levels are positively associated with the Gleason score and BCR (Ihlaseh-Catalano et al., 2013; Gomes et al., 2014). In contrast, STEAP1 was upregulated in the normal human endometrium and downregulated in most endometrial cancer tissue specimens. STEAP1 downregulation is associated with the degree of malignancy, tumor differentiation, and lymph node metastasis in endometrial cancer. Furthermore, the prognosis of patients with endometrial cancer with high STEAP1 expression levels was significantly better than that of patients with low STEAP1 expression levels (Sun et al., 2019). Moreover, STEAP1 was downregulated in breast cancer tissues and negatively correlated with the TNM stage, tumor grade, and lymph node metastasis in breast cancer patients. Kaplan–Meier analysis previously revealed that breast cancer patients with low STEAP1 expression levels tended to have a poor prognosis (Xie et al., 2019). Additionally, Zhuang et al. first reported that STEAP1 is upregulated in lung cancer tissue; however, no further assessment was conducted (Zhuang et al., 2015). This study shows that STEAP1 is upregulated in LUAD tissue, which is associated with the TNM stage and LNM. These results were further confirmed using the RNA-seq data from TCGA and GEO databases. Together, these results suggest that STEAP1 is a potential prognostic biomarker for LUAD.

The cell cycle is closely associated with the tumorigenesis and cancer progression. For instance, Wang et al. reported that the p53/p21 signaling pathway potentially inhibits SPAG5 expression, thereby regulating the proliferation and migration of LUAD cells (Wang T. et al., 2019). Similarly, Tan et al. reported that TNKS1BP1 was upregulated in LUAD and promoted cell cycle transformation (S phase accumulation and M phase decrease), and was associated with a poor prognosis of cancer patients. Furthermore, they reported that TNKS1BP1 regulates genome stability by influencing homologous recombination (Tan et al., 2017). Through Metascape analysis, this study shows that STEAP1 is involved in processes closely associated with tumorigeneses, such as cell division, cytokine production, cytokine signaling, and DNA replication. Further, KEGG analysis indicated that genes co-expressed with STEAP1 were involved in the cell cycle, the IL-17 signaling pathway, ECM receptor interaction, apoptosis, the NF-kB signaling pathway, and the TNF signaling pathway. These results were further validated via GSEA, which revealed that STEAP1 upregulation was associated with homologous recombination, the p53 signaling pathway, cell cycle, DNA replication, apoptosis, and the Toll-like receptor signaling pathway. STEAP1 silencing reportedly inhibited the activity and proliferation of LNCaP cells and induced apoptosis in prostate cancer by affecting p53, p21, Bax, Bax/Bcl-2, Caspase-9, FasL, Caspase-8, and Caspase-3 (Gomes et al., 2018). Furthermore, STEAP1 silencing inhibited proliferation, clonal formation, and invasion *in vitro* and *in vivo* in Ewing’s tumor. Moreover, STAT1 activation would be inhibited upon STEAP1 knockout (Grunewald et al., 2012b). Interference of STEAP1 inhibited proliferation but promoted apoptosis and increased the production of reactive oxygen species (ROS) in colorectal cancer cells. Considering the potential underlying mechanism, STEAP1 silencing inhibited the expression of nuclear erythroid 2-related factor (NRF2) in colorectal cancer cells (Nakamura et al., 2019) but promoted cell cycle transformation (G-S phase), thereby promoting cell proliferation and enhancing cell invasion, migration, and tumorigenesis in nude mice. Furthermore, STEAP1 knockdown inhibited the expression of E-cadherin but promoted the expression of N-cadherin, Vimentin, Snail, Slug, Twist, MMP2, and MMP9 (Sun et al., 2019). Therefore, based previous and the present data, we believe that STEAP1 potentially promotes the occurrence and progression of LUAD through its effects on the cell cycle, DNA replication, and apoptosis.

The 10 hub genes identified herein in the PPI network were closely associated with the cell cycle and potentially play a pivotal role in LUAD pathogenesis. CDK1 upregulation has been reported in LUAD and is reportedly associated with a poor patient prognosis (Liu W. T. et al., 2018). Further, nadroparin potentially inhibits the growth of LUAD-derived A549 and CALU1 cells by downregulating CDK1 (Carmazzi et al., 2012). In a related study, inhibition of UBE2C, an upregulated marker of esophageal squamous cell carcinoma, inhibited the growth of esophageal squamous cell carcinoma by modulating the expression of CCNB1, which was one of the ten hub genes identified in our previous study (Palumbo et al., 2016). CCNA2 is reportedly upregulated in NSCLC tissues and associated with a poor patient prognosis (Ko et al., 2013). Furthermore, CCNB2, MAD2L1, BUB1, and NCAPG are significantly upregulated in hepatocellular carcinoma (HCC) tissues and are implicated in the growth and metastasis of HCC cells (Li et al., 2017, 2019; Xu et al., 2017; Liu K. et al., 2018). BIRC5 is reportedly upregulated in ovarian cancer cells, and its inhibition impeded the growth and migration of ovarian cancer cells (Wang et al., 2018). KIF4A, another hub gene identified herein, is reportedly upregulated in colorectal cancer tissues and cell lines and was significantly associated with patient clinicopathological features including OS and DFS herein. Interestingly, KIF4A exerted its tumorigenic effects by regulating the cell cycle and cell proliferation (Hou et al., 2018). Together, these results show that the hub genes identified herein play a role in the occurrence and progression of numerous tumors by regulating various signaling pathways. These results also support our hypothesis that upregulation of CDK1, UBE2C, CCNA2, CCNB1, CCNB2, MAD2L1, BIRC5, BUB1, NCAPG, and KIF4A is potentially associated with a poor prognosis of LUAD patients. However, we could not unravel the complex interactions among these hub genes and STEAP1, and our results warrant further experimental verification to confirm their interplay in LUAD.

## Conclusion

The present results show that STEAP1 is upregulated in LUAD and is associated with the clinicopathological features and prognosis of LUAD patients. Moreover, STEAP1 potentially promotes the occurrence and progression of LUAD by regulating homologous recombination, the p53 signaling pathway, cell cycle, DNA replication, and apoptosis, thus serving as a potential prognostic biomarker for LUAD.

## Data Availability Statement

Publicly available datasets were analyzed in this study. The datasets generated for this study can be accessed in The Cancer Genome Atlas (TCGA) database [https://portal.gdc.cancer.gov/projects/TCGA-LUAD(HTSeq-FPKM)] and NCBI Gene Expression Omnibus (GEO), GSE2514 (GPL8300), and GSE10072 (GPL96) (http://www.ncbi.nlm.nih.gov/geo).

## Ethics Statement

The study involving human participants was reviewed and approved by the Ethics Committee of the Affiliated Hospital of Zunyi Medical University. The patients/participants provided written informed consent to participate in this study.

## Author Contributions

All authors contributed toward completing this study, read, and approved the final manuscript. GX, QG, and H-LL conceived the research topic, made the research plan, and directed the implementation of the experiments. QG and XK contributed equally to analyze the data and draft the manuscript. ZL, W-LG, and S-XF assisted in data analysis and drafting the manuscript. CC, Y-XS, and HH involved in the management of lung cancer samples and the collection of related data.

## Conflict of Interest

The authors declare that the research was conducted in the absence of any commercial or financial relationships that could be construed as a potential conflict of interest. The reviewer MC and handling Editor declared their shared affiliation at the time of review.

## Footnotes

1 https://portal.gdc.cancer.gov/

2 https://string-db.org/

3 http://gepia.cancer-pku.cn/

## References

[B1] Barroca-Ferreira J. Pais J. P. Santos M. M. Goncalves A. M. Gomes I. M. Sousa I. (2018). Targeting STEAP1 protein in human cancer: current trends and future challenges. *Curr. Cancer Drug Targets* 18 222–230. 10.2174/1568009617666170427103732 28460619

[B2] Bray F. Ferlay J. Soerjomataram I. Siegel R. L. Torre L. A. Jemal A. (2018). Global cancer statistics 2018: GLOBOCAN estimates of incidence and mortality worldwide for 36 cancers in 185 countries. *CA Cancer J. Clin.* 68 394–424. 10.3322/caac.21492 30207593

[B3] Carmazzi Y. Iorio M. Armani C. Cianchetti S. Raggi F. Neri T. (2012). The mechanisms of nadroparin-mediated inhibition of proliferation of two human lung cancer cell lines. *Cell Prolif.* 45 545–556. 10.1111/j.1365-2184.2012.00847.x 23106301 PMC6495835

[B4] Colling R. Bancroft H. Langman G. Soilleux E. (2019). Fully automated real-time PCR for EGFR testing in non-small cell lung carcinoma. *Virchows Arch.* 474 187–192. 10.1007/s00428-018-2486-y 30470932 PMC6349793

[B5] El-Zein R. A. Abdel-Rahman S. Santee K. J. Yu R. Shete S. (2017). Identification of small and non-small cell lung cancer markers in peripheral blood using cytokinesis-blocked micronucleus and spectral karyotyping assays. *Cytogenet Genome Res.* 152 122–131. 10.1159/000479809 28898877

[B6] Feng S. Zheng J. Du X. Tan Y. Yang H. Zhang H. (2013). Human papillomavirus was not detected by PCR using multiple consensus primer sets in esophageal adenocarcinomas in Chinese patients. *J. Med. Virol.* 85 1053–1057. 10.1002/jmv.23468 23588731

[B7] Gomes I. M. Rocha S. M. Gaspar C. Alvelos M. I. Santos C. R. Socorro S. (2018). Knockdown of STEAP1 inhibits cell growth and induces apoptosis in LNCaP prostate cancer cells counteracting the effect of androgens. *Med. Oncol.* 35:40. 10.1007/s12032-018-1100-0 29464393

[B8] Gomes I. M. Santos C. R. Socorro S. Maia C. J. (2013). Six transmembrane epithelial antigen of the prostate 1 is down-regulated by sex hormones in prostate cells. *Prostate* 73 605–613. 10.1002/pros.22601 23060075

[B9] Gomes I. M. Arinto P. Lopes C. Santos C. R. Maia C. J. (2014). STEAP1 is overexpressed in prostate cancer and prostatic intraepithelial neoplasia lesions, and it is positively associated with Gleason score. *Urol. Oncol.* 32:53.e23-59.23. 10.1016/j.urolonc.2013.08.028 24239460

[B10] Grunewald T. G. Bach H. Cossarizza A. Matsumoto I. (2012a). The STEAP protein family: versatile oxidoreductases and targets for cancer immunotherapy with overlapping and distinct cellular functions. *Biol. Cell.* 104 641–657. 10.1111/boc.201200027 22804687

[B11] Grunewald T. G. Diebold I. Esposito I. Plehm S. Hauer K. Thiel U. (2012b). STEAP1 is associated with the invasive and oxidative stress phenotype of Ewing tumors. *Mol. Cancer Res.* 10 52–65. 10.1158/1541-7786 22080479

[B12] Grunewald T. G. Ranft A. Esposito I. da Silva-Buttkus P. Aichler M. Baumhoer D. (2012c). High STEAP1 expression is associated with improved outcome of Ewing’s sarcoma patients. *Ann. Oncol.* 23 2185–2190. 10.1093/annonc/mdr605 22317770

[B13] Hasan N. Kumar R. Kavuru M. S. (2014). Lung cancer screening beyond low-dose computed tomography: the role of novel biomarkers. *Lung* 192 639–648. 10.1007/s00408-014-9636-z 25108403

[B14] Hou P. F. Jiang T. Chen F. Shi P. C. Li H. Q. Bai J. (2018). KIF4A facilitates cell proliferation via induction of p21-mediated cell cycle progression and promotes metastasis in colorectal cancer. *Cell Death Dis.* 9:477. 10.1038/s41419-018-0550-9 29706624 PMC5924760

[B15] Ihlaseh-Catalano S. M. Drigo S. A. de Jesus C. M. Domingues M. A. Trindade Filho J. C. de Camargo J. L. (2013). STEAP1 protein overexpression is an independent marker for biochemical recurrence in prostate carcinoma. *Histopathology* 63 678–685. 10.1111/his.12226 24025158

[B16] Kassem K. Shapiro M. Gorenstein L. Patel K. Laird C. (2019). Evaluation of high-risk pulmonary nodules and pathologic correlation in patients enrolled in a low-dose computed tomography (LDCT) program. *J. Thorac. Dis.* 11 1165–1169. 10.21037/jtd.2019.04.31 31179058 PMC6531697

[B17] Kleczko E. K. Kwak J. W. Schenk E. L. Nemenoff R. A. (2019). Targeting the complement pathway as a therapeutic strategy in lung cancer. *Front. Immunol.* 10:954. 10.3389/fimmu.2019.00954 31134065 PMC6522855

[B18] Ko E. Kim Y. Cho E. Y. Han J. Shim Y. M. Park J. (2013). Synergistic effect of Bcl-2 and cyclin A2 on adverse recurrence-free survival in stage I non-small cell lung cancer. *Ann. Surg. Oncol.* 20 1005–1012. 10.1245/s10434-012-2727-2 23115005

[B19] Lee C. H. Chen S. L. Sung W. W. Lai H. W. Hsieh M. J. Yen H. H. (2016). The prognostic role of STEAP1 expression determined via immunohistochemistry staining in predicting prognosis of primary colorectal cancer: a survival analysis. *Int. J. Mol. Sci.* 17:E592. 10.3390/ijms17040592 27104516 PMC4849046

[B20] Li R. Jiang X. Zhang Y. Wang S. Chen X. Yu X. (2019). Cyclin B2 overexpression in human hepatocellular carcinoma is associated with poor prognosis. *Arch. Med. Res*. 50 10–17. 10.1016/j.arcmed.2019.03.003 31101236

[B21] Li X. Gu G. Soliman F. Sanders A. J. Wang X. Liu C. (2018). The evaluation of durative transfusion of endostar combined with chemotherapy in patients with advanced non-small cell lung cancer. *Chemotherapy* 63 214–219. 10.1159/000493098 30347389

[B22] Li Y. Bai W. Zhang J. (2017). MiR-200c-5p suppresses proliferation and metastasis of human hepatocellular carcinoma (HCC) via suppressing MAD2L1. *Biomed Pharmacother.* 92 1038–1044. 10.1016/j.biopha.2017.05 28609841

[B23] Liu K. Li Y. Yu B. Wang F. Mi T. Zhao Y. (2018). Silencing non-SMC chromosome-associated polypeptide G inhibits proliferation and induces apoptosis in hepatocellular carcinoma cells. *Can. J. Physiol. Pharmacol.* 96 1246–1254. 10.1139/cjpp-2018-0195 30089216

[B24] Liu W. T. Wang Y. Zhang J. Ye F. Huang X. H. Li B. (2018). A novel strategy of integrated microarray analysis identifies CENPA, CDK1 and CDC20 as a cluster of diagnostic biomarkers in lung adenocarcinoma. *Cancer Lett.* 425 43–53. 10.1016/j 29608985

[B25] Maemura K. Watanabe K. Ando T. Hiyama N. Sakatani T. Amano Y. (2018). Altered editing level of microRNAs is a potential biomarker in lung adenocarcinoma. *Cancer Sci.* 109 3326–3335. 10.1111/cas.13742 30022565 PMC6172074

[B26] Molina J. R. Yang P. Cassivi S. D. Schild S. E. Adjei A. A. (2008). Non-small cell lung cancer: epidemiology, risk factors, treatment, and survivorship. *Mayo Clin. Proc.* 83 584–594. 10.4065/83.5.584 18452692 PMC2718421

[B27] Nakamura H. Takada K. Arihara Y. Hayasaka N. Murase K. Iyama S. (2019). Six-transmembrane epithelial antigen of the prostate 1 protects against increased oxidative stress via a nuclear erythroid 2-related factor pathway in colorectal cancer. *Cancer Gene Ther.* 26 313–322. 10.1038/s41417-018-0056-8 30401882

[B28] Palumbo A. Da Costa N. M. De Martino M. Sepe R. Pellecchia S. de Sousa V. P. (2016). UBE2C is overexpressed in ESCC tissues and its abrogation attenuates the malignant phenotype of ESCC cell lines. *Oncotarget* 7 65876–65887. 10.18632/oncotarget.11674 27588470 PMC5323199

[B29] Sun J. Ji G. Xie J. Jiao Z. Zhang H. Chen J. (2019). Six-transmembrane epithelial antigen of the prostate 1 is associated with tumor invasion and migration in endometrial carcinomas. *J. Cell Biochem*. 10.1002/jcb.28393 [Epub ahead of print]. 30714206

[B30] Szklarczyk D. Franceschini A. Wyder S. Forslund K. Heller D. Huerta-Cepas J. (2015). STRING v10: protein-protein interaction networks, integrated over the tree of life. *Nucleic Acids Res.* 43 D447–D452. 10.1093/nar/gku1003 25352553 PMC4383874

[B31] Tan W. Guan H. Zou L. H. Wang Y. Liu X. D. Rang W. Q. (2017). Overexpression of TNKS1BP1 in lung cancers and its involvement in homologous recombination pathway of DNA double-strand breaks. *Cancer Med.* 6 483–493. 10.1002/cam4.995 28058814 PMC5313643

[B32] Tang Z. Li C. Kang B. Gao G. Li C. Zhang Z. (2017). GEPIA: a web server for cancer and normal gene expression profiling and interactive analyses. *Nucleic Acids Res.* 45 W98–W102. 10.1093/nar/gkx247 28407145 PMC5570223

[B33] Wang B. Li X. Zhao G. Yan H. Dong P. Watari H. (2018). miR-203 inhibits ovarian tumor metastasis by targeting BIRC5 and attenuating the TGFβ pathway. *J. Exp. Clin. Cancer Res.* 37:235. 10.1186/s13046-018-0906-0 30241553 PMC6150978

[B34] Wang F. Chan L. W. Tsui N. B. Wong S. C. Siu P. M. Yip S. P. (2015). Coexpression Pattern Analysis of NPM1-Associated Genes in Chronic Myelogenous Leukemia. *Biomed Res Int.* 2015:610595. 10.1155/2015/610595 25961029 PMC4413041

[B35] Wang L. Shi J. Huang Y. Liu S. Zhang J. Ding H. (2019). A six-gene prognostic model predicts overall survival in bladder cancer patients. *Cancer Cell Int.* 19:229. 10.1186/s12935-019-0950-7 31516386 PMC6729005

[B36] Wang T. Li K. Song H. Xu D. Liao Y. Jing B. (2019). p53 suppression is essential for oncogenic SPAG5 upregulation in lung adenocarcinoma. *Biochem. Biophys. Res. Commun.* 513 319–325. 10.1016/j.bbrc.2019.03.198 30955859

[B37] Wu H. Zhang J. (2018). Decreased expression of TFAP2B in endometrial cancer predicts poor prognosis: a study based on TCGA data. *Gynecol. Oncol.* 149 592–597. 10.1016/j.ygyno.2018.03.057 29602546

[B38] Xie J. Yang Y. Sun J. Jiao Z. Zhang H. Chen J. (2019). STEAP1 inhibits breast cancer metastasis and is associated with epithelial-mesenchymal transition procession. *Cl.in Breast Cancer* 19 e195–e207. 10.1016/j.clbc.2018.08.010 30253922

[B39] Xu B. Xu T. Liu H. Min Q. Wang S. Song Q. (2017). MiR-490-5p suppresses cell proliferation and invasion by targeting BUB1 in hepatocellular carcinoma cells. *Pharmacology* 100 269–282. 10.1159/000477667 28810242

[B40] Yang G. Zhang Y. Yang J. (2019). A five-microRNA signature as prognostic biomarker in colorectal cancer by bioinformatics analysis. *Front. Oncol.* 9:1207. 10.3389/fonc.2019.01207 31799184 PMC6863365

[B41] Zhang J. N. Qin X. Liu X. G. Ma X. N. Huang Z. P. Zhang W. (2020). Identification of abnormally expressed genes and their roles in invasion and migration of hepatocellular carcinoma. *Aging* 12 10.18632/aging.102727 32017706

[B42] Zhou Y. Zhou B. Pache L. Chang M. Khodabakhshi A. H. Tanaseichuk O. (2019). Metascape provides a biologist-oriented resource for the analysis of systems-level datasets. *Nat. Commun.* 10:1523. 10.1038/s41467-019-09234-6 30944313 PMC6447622

[B43] Zhuang X. Herbert J. M. Lodhia P. Bradford J. Turner A. M. Newby P. M. (2015). Identification of novel vascular targets in lung cancer. *Br. J. Cancer* 112 485–494. 10.1038/bjc.2014.626 25535734 PMC4453649

