# Supplementary material for: Evaluation of the Prognostic Value of STEAP1 in Lung Adenocarcinoma and Insights Into Its Potential Molecular Pathways via Bioinformatic Analysis
Source: Front Genet. 2020 Mar 20;11:242. doi: 10.3389/fgene.2020.00242 (PMC7099762; doi:10.3389/fgene.2020.00242)
Supplement: Supplementary file 1 [file Table_1.DOCX]

Supplementary Material

# Table S1:STEAP1 co-expression gene

| Cor type | Gene |
| --- | --- |
| Positive correlation | STEAP2, CCDC71L, C15orf48, ARNTL2, CALU, ERO1A, ACTR3, SRPX2, PLAU, TPM4, FAM3C, MMD, TGFBI, NAMPT, SPHK1, RND3, CLDN12, FOSL1, GPX8, CTHRC1, CXCL8, SPATS2L, GREM1, TPBG, SLC2A1, GFPT2, VRK2, PLOD2, GPC6, ADGRF4, TNFAIP6, CCDC90B, GJB2, LAMC2, PLSCR1, PCDH7, MB21D1, GPR87, RALB, C10orf55, FBXO32, CD109, NMI, ELK3, UBE2F, CCL26, RIPK2, AMFR, MARCKS, MDFIC, DBF4, BEND6, DSE, GPR84, UBE2E3, HILPDA, SEMA3C, CCDC109B, IL15RA, CORO1C, PSMC2, BCL10, ANLN, FHL2, PLPP4, GBP1, AIM2, MAD2L1, SHCBP1, KLHL5, TNFSF4, GOLT1B, SULF1, LOX, RPL39L, B3GALNT1, ARPC1A, CCL11, CCNA2, SLC16A3, KIAA1524, TES, NCK1, CPD, PGK1, CCL7, MTPN, RRM2, TMEM45A, CEP55, S100A8, ANKIB1, LOXL2, PGM2, ADAM12, VEGFC, SPOCD1, BZW1, ARPC2, MMP14, ORC5MSANTD3, PMAIP1, CDCP1, EPHB2,RAB32, ADM, RGS20, NMD3, WISP1, FRMD6, SKA3, SLC2A5, TMEM158, LDHA, RCN1, ITGA5, CLMP, RHOC, B3GNT5, HAPLN3, ANXA2, CENPW, LGALS1, NTAN1, YWHAG, GPN1, CDKN3, EGLN3, KPNA2, CLEC2B, ARMC10, C7orf49, IKBIP, BIRC5, RELB, SNAI1, S100A9, PLIN3, POT1, ZNF267, FAP, POSTN, ZFAND2A, DCBLD2, PLAUR, ITGAV, RHOF, ADA, PNPLA8, CENPK, VOPP1, SKAP2,TMEM171, GJB3, IFI16, FAM220A, PDK3, FMO1, INPP4B, SRPRB, ELOVL6, SEC23A, CAPZA1, CD274, SOD2, UBE2C, RAD51, SFRP2, PKIB, CDK1, EPSTI1, TRIP13, TUBA1C, TWIST1, DUS4L, RARRES1, EIF5A2, RAD51AP1, CDC6, PPP1R18, PRR11 ,PDCD1LG2UBA6, CDCA5, MILR1, MSC, HIF1A, PSMB9, PRDM8, MELK, TWSG1, HPRT1, MT1X, BCL2A1, DTX2, MTHFD2, SEMA7A, GZMB, NCEH1, ADAM19, CDH3, PRR16, CTSB, NUP54, ASF1B, SPAG4, LAYN, CCNE1, BCAR3, HJURP, CLDND1, ACOT9, COL11A1, TK1, TDO2, DLGAP5, SAA1, IL1R2, CENPU, CXCL10, GLIPR1, MMP3, NGEF, TNFRSF9, SGOL2, IL15, HS3ST3A1, CCNB1, ERGIC2, HMMR, CENPN, GYG1, CDA, LYPD3, PTPRH, CCL8, NCAPG, OSBPL3, CKAP2L, PALLD, FRMD5, TPX2, CTSL, TMEM55A, DCUN1D5, PSMD14, KCTD5, KIF4A, CTSV, SEMA3A, CENPA, KDELC2, CHCHD3, UCHL3, DDIAS, LRRC42, PLEK2, TAP1, CCNB2, CDK6, VCAM1, FAM83A, PRSS23, COLGALT1, DEPDC1, TUBB6, STX1A, ZC3H15, CENPI, RECQL, PDLIM5, RAP2C, BUB1, ADGRG3, SULF2, EIF2S2, DAPP1, LXN, IL23A, MT2A, MYBL2, COL3A1, FHOD3, SMS, CKLF, FAM83D, SPC25, TTK, C12orf4, PGM2L1, UBE2L6, NPTN, DUSP14, THBS2, COPS8, BIRC3, FAM26F. |
| Negative correlation | UNC13B, STXBP1, CACNA2D2, ADGRD1, SELENBP1, KCNJ11, MOAP1, GPD1L, ATP8A1, PPP1R13B, KLF15, ESYT3, ADCK3, PRICKLE4, LIMD1, PLA2G4F, B3GNT8, BTBD9, AK1, SOX13, RFTN1, FRAT1, C16orf89, RAP1GAP, PARM1, CNTROB, SLC25A4, PLA2G1B, CHIA, RHOBTB2, ARHGEF2, DLC1, SFTA3, KIF12, ATOH8, OVGP1, SUSD2, MARC1, CYP4B1, IRX3, LDB1, ZNF385B, ALDH6A1, JPH1, ALDH5A1, NEDD4L, CIT, DAPK2, ZBED3, CABLES1, ACADSB, PLXNA2, ABCA3, CNNM3, IRX5, GRAMD2, CIRBP, MYLIP, ACOXL, ACSS1, NISCH, BCAM, FBXW4, FRS3, VAMP2, SLC27A3, NECAB3, SFTPB, AMIGO1, ZBTB18, NICN1, IL17RE, ST6GALNAC4, TMEM125, NEK8, BCAT2, CRY2, AQP7, ZNF778, IVD, TAPT1, RALGPS1, TFCP2L1 FAAH, ATP11A, PGC, HUNK, CGNL1, PNPLA7, MTURN, CRTC1,PLXNB1, FAM184A, TNS2, DCAF8, ZBTB4, PMM1, ZNF219, MRPS25, PHACTR1, VIPR1, IFT140, TMEM91, ABHD14A, GGTLC1, SLC22A31, GJB1, RCOR3, ALDH3A2, PBXIP1, DAPK1, PEBP1, SUOX, EFCC1, WDR6, BTG2, ESRP2, SNX30, NR0B2, TMEM63B, SNX25, C1orf116, MAGI1, LPL, TDRD10, DAAM2, ZNF76, EPHX1, RPH3AL, HSDL2, GNMT,FAM117A, MAOA, ANKRD29, SHE, LARGE, ADGRF5, FOLR1, PLA2G12B, AATK, TMEM163, ABCC6, ATP1B2, HLF, CPB2, FOXP4, ZDHHC16, COL4A4, MFSD2A, FAM189A2, P3H2, HNF1B, AP2A2, ADHFE1, PLA2G6, GKAP1, AARD, TPPP, C1orf210, SLC47A1, OIT3, ADI1, MBIP, MMP15, ST3GAL5, GMPR, ROGDI, BTNL9, GPRC5C, BCOR, TMEM80, POLR3H, GREB1, ZBTB22, SCNN1B, C9orf152, ZNF444, DUOX1, TAF8, PPP2R5A, SLC48A1, SEMA4A, RAB40B, ARRB1, COL4A3, CPT2, SLAIN1, KLC4, NKX2-1, C5orf38. |

# 
